# Management of ERCP- and EUS-related duodenal perforations using over-the-scope clips

**DOI:** 10.1016/j.igie.2022.12.003

**Published:** 2023-02-21

**Authors:** Ahmad M. Al-Taee, Jonathan Cohen, Yunseok Namn, Gregory B. Haber

**Affiliations:** Center for Therapeutic Advanced Endoscopy and Innovation, Division of Gastroenterology and Hepatology, NYU Langone School of Medicine, New York, New York, USA

## Abstract

EUS and ERCP are widely used for the evaluation and management of various pancreatobiliary conditions. They are generally regarded as safe procedures. Perforation is a rare but life-threatening adverse event of EUS and ERCP. Here we present 3 cases of ERCP- and EUS-related perforations that were successfully managed with over-the-scope clips (OTSCs). This work highlights the importance of early recognition and management of post-ERCP and -EUS perforations to ensure the best outcome possible. OTSCs have expanded the ability to close larger perforations and fistulas and could be considered a first-line tool for endoscopic closure of ERCP- and EUS-related perforations.

ERCP and EUS are generally regarded as safe procedures. Although rare, perforation is one of the most serious and dreaded adverse events of both procedures. Immediate recognition and endoscopic closure, if at all possible, is of paramount importance to successful management. The over-the-scope clip (OTSC) device is a novel endoscopic closure system that has expanded the ability to close perforations and fistulas. Here we present 3 cases that were successfully managed by endoscopic closure using OTSCs.

## Case Descriptions

### Case 1

A 57-year-old woman with a remote history of cholecystectomy was referred to us for management of symptomatic choledocholithiasis. She previously underwent 2 ERCPs with incomplete stone extraction and placement of a plastic biliary stent.

**Figure 1:**
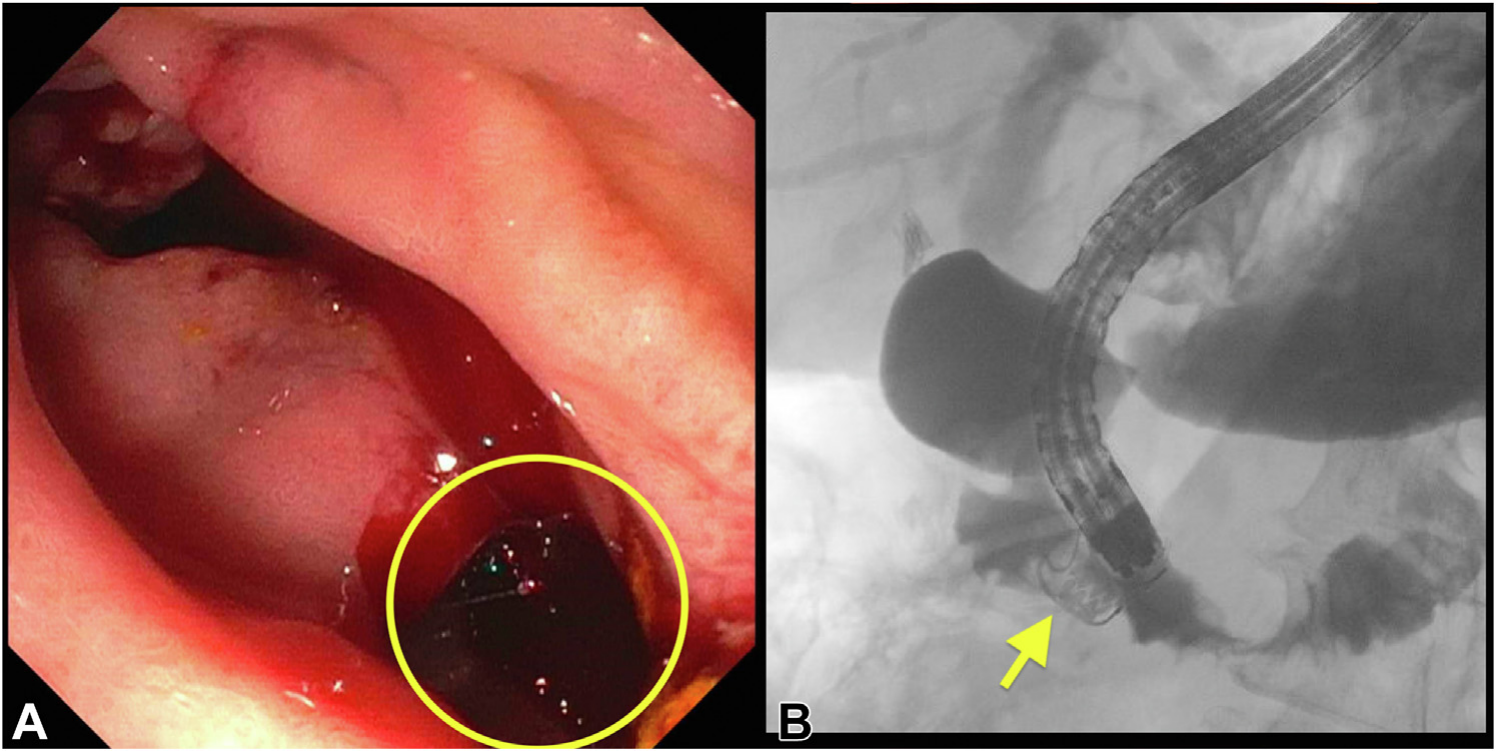
**A,** ERCP performed for choledocholithiasis. On scope withdrawal, a lateral duodenal perforation was noted (*yellow circle*) and was successfully closed using an over-the-scope clip. **B,** Subsequent contrast injection over the clip (*yellow arrow*) revealed no extravasation.

We performed a repeat ERCP where the biliary stent was removed and the biliary sphincterotomy appeared small. Moreover, there was resistance to passing an 8.5-mm balloon catheter across the papilla. Therefore, an extension sphincterotomy as well as a sphincteroplasty using a 20-mm balloon dilator was also performed. The biliary tree was swept using a combination of a 20-mm extraction balloon and extraction basket, and many stones and sludge were removed. A 20-mm full-thickness defect was noted on the lateral wall of the second part of the duodenum. The duodenoscope was withdrawn. A therapeutic upper endoscope was fitted with a 12/6T OTSC and was advanced to the second part of the duodenum. The cap was maneuvered over the defect. A twin grasper was used to grasp the 2 edges of the defect. The OTSC was successfully placed over the defect, and sequential contrast injection over the clip revealed no extravasation on fluoroscopy (Fig. 1 and Video 1, available online at www.igiejournal.org). A nasogastric tube was successfully placed, and intravenous antibiotics were administered.

The patient was admitted to the hospital for observation. An upper GI series the following day showed no extravasation. She tolerated gradually advancing the diet and was discharged home 2 days later.

### Case 2

A 92-year-old man with a history of recently diagnosed pancreatic head adenocarcinoma presented for ERCP for biliary decompression. For evaluation of elevated liver enzymes, he previously underwent abdominal CT that showed a pancreatic head mass with associated diffuse upstream biliary ductal dilation, consistent with distal malignant biliary obstruction.

**Figure 2:**
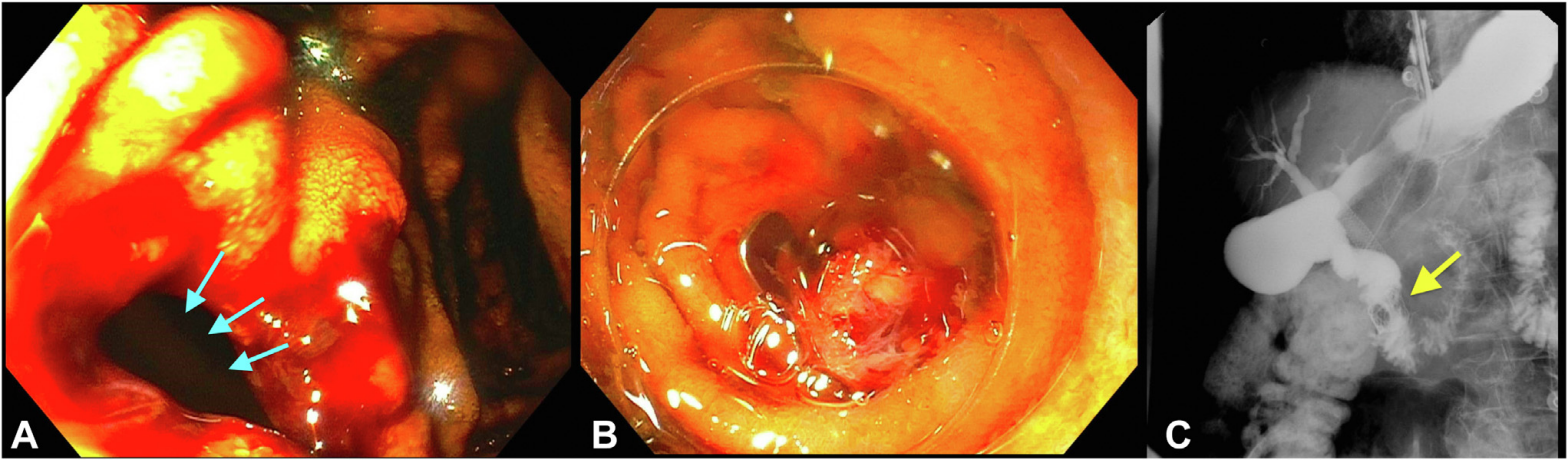
**A,** A lateral duodenal wall perforation was noted during ERCP performed for decompression of malignant biliary obstruction (*blue arrows*). **B,** Successful closure was achieved using an over-the-scope clip. **C,** An upper GI series the following day showed no contrast extravasation. The *yellow arrows* shows the previously deployed over-the-scope clip.

After successful biliary cannulation, the cholangiogram showed a malignant-appearing stricture in the distal common bile duct with diffuse upstream biliary ductal dilation. A partially covered metal biliary stent was placed across the stricture with copious drainage of dark bilious fluid. On withdrawal of the duodenoscope, a 20-mm defect was identified on the lateral wall of the second portion of the duodenum. The duodenoscope was withdrawn. A therapeutic upper endoscope was fitted with a 12/6T OTSC and was advanced to the second portion of the duodenum. The OTSC was successfully deployed over the perforation site with excellent approximation of the defect margins using twin graspers and light endoscopic suction. Subsequent injection of full-strength contrast into the duodenal lumen showed no evidence of extravasation on fluoroscopy (Fig. 2).

The patient was admitted to the hospital for observation and tolerated gradually advancing the diet. An upper GI series the following day showed no extravasation. He was discharged home 3 days after the ERCP.

### Case 3

A 74-year-old woman with a history of atrial fibrillation on warfarin was referred for further evaluation of a periampullary lesion. For evaluation of persistent abdominal pain and nausea, she previously underwent an abdominal CT that showed a 1.4-cm periampullary enhancing soft tissue lesion with associated intrahepatic and extrahepatic biliary ductal dilatation. She had a pacemaker that was not compatible with magnetic resonance imaging. Therefore, she was referred to us for upper EUS.

**Figure 3:**
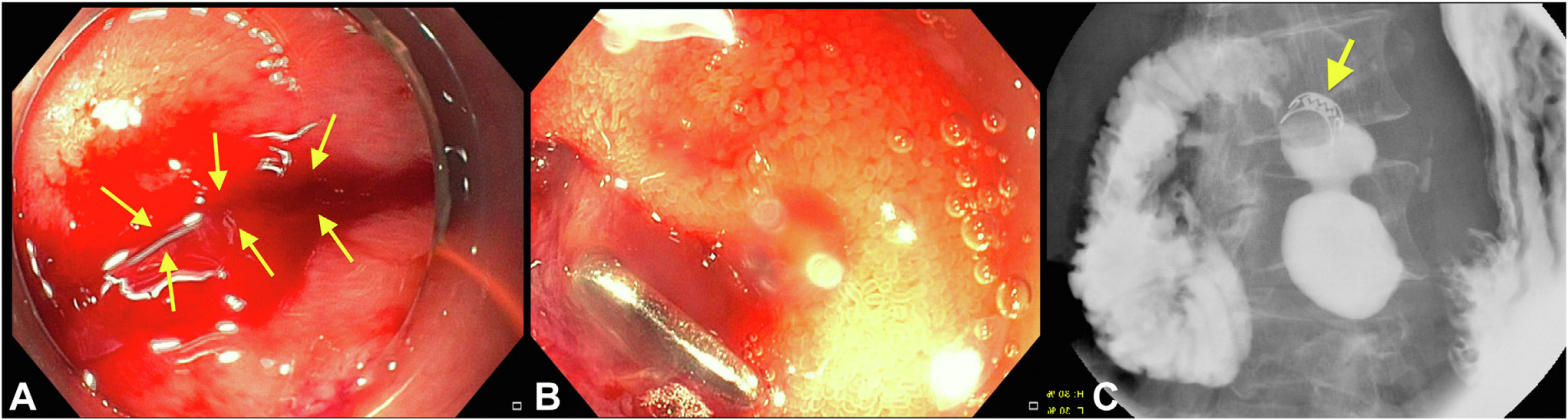
**A,** A 74-year-old woman presented for evaluation of a periampullary lesion noted on cross-sectional imaging. On attempting to pass the radial echoendoscope across the duodenal sweep, a duodenal bulb perforation was noted (*yellow arrows*). **B,** The perforation was successfully closed using an over-the-scope clip. **C,** An upper GI series showed no evidence of extravasation.

Endoscopic examination of the esophagus and stomach was unremarkable. A 10-mm nonbleeding diverticulum was found in the second portion of the duodenum. The radial echoendoscope was advanced into the duodenal bulb. A 15-mm perforation was noted in the duodenal bulb after attempting to pass the echoendoscope across the duodenal sweep. The echoendoscope was withdrawn. A therapeutic upper endoscope was fitted with a 12/6T OTSC and was advanced to the second part of the duodenum. The cap was maneuvered over the defect. A twin grasper was used to grasp the 2 edges of the defect. The OTSC was successfully deployed to close the defect (Fig. 3). Sequential injection of contrast over the clip revealed no extravasation on fluoroscopy. A nasogastric tube was successfully placed, and intravenous antibiotics were administered.

The patient was admitted to the hospital for observation. An upper GI series showed no evidence of extravasation. She tolerated gradually advancing the diet and was discharged home 4 days later.

## Discussion

Early recognition and treatment of ERCP- and EUS-related perforations are the most important factors determining clinical outcome.^1^ This is facilitated by slow withdrawal and careful inspection during scope withdrawal. Most cases can be managed conservatively if immediate endoscopic closure is successful.^1^, ^2^, ^3^, ^4^ Otherwise, surgical repair might be required in up to 50% of perforations and is more likely in cases with type 1 duodenal perforation.^5^ Patients with EUS- or ERCP-related duodenal perforation should be kept fasting while receiving intravenous hydration, nasogastric suction, and intravenous antibiotics. Early surgical consultation should be obtained.

The main endoscopic options for closure include through-the-scope clips,^6^^,^^7^ OTSCs,^8^^,^^9^ and endoscopic suturing.^10^ We have compiled a list of the previously published reports on endoscopic closure of nonampullary duodenal perforations in Table 1. Through-the-scope clips are usually reserved for closure of perforations less than 10 mm in size. The OTSC device (Ovesco Endoscopy, Tübingen, Germany) is a novel endoscopic closure system that has been used in closure of perforations and fistulas^11^ and for treatment of GI bleeding.^12^ The OTSC with full-thickness grasping capability is generally preferred over through-the-scope clips for closure of larger perforations between 10 and 25 mm.^13^ The OTSC system should be considered before surgical repair for closure of ERCP- and EUS-related perforations. Endoscopic suturing using the Overstitch device (Apollo Endosurgery, Austin, Tex, USA) is an alternative closure method and is the preferred method for perforations that are too large to be closed by OTSCs.^14^^,^^15^ We typically do not remove these clips as, like other clips, OTSCs tend to fall off after a certain period of time, and such a timespan is usually sufficient for resolution of a pathology like an acute perforation.

**Table 1:** Review of published case reports on endoscopic closure of nonampullary duodenal perforations

Study	Age (y)	Gender	Procedure	Perforation type and size	Closure method	Need for surgical repair	Hospitalization outcome
Lee et al^6^	80	F	ERCP	D1, >10 mm	TTSC	No	Discharge after 12 days
	72	F	ERCP	Lateral wall of D2, >10 mm	TTSC	No	Discharge after 10 days
	69	F	ERCP	Lateral wall of D2, >10 mm	TTSC	No	Discharge after 10 days
	63	M	ERCP	Lateral wall of D2, >10 mm	TTSC	No	Discharge after 27 days
Donatelli et al^8^	66	M	ERCP	D1, >20 mm	OTSC	No	Discharge after 7 days
Mangiavillano et al^16^	Not reported	Not reported	EUS	Not reported	OTSC	No	Not reported
	Not reported	Not reported	ERCP	Not reported	OTSC	Yes	Not reported
Nakagawa et al^17^	88	M	ERCP	Lateral wall of D2, 30 mm	Endoloops and endoclips	No	Not reported
Hyun et al^14^	83	M	Upper endoscopy	Duodenal sweep, ≥20 mm	Endoscopic suturing	No	Not reported
Lee et al^15^	73	F	ERCP	D1, size not reported	Endoscopic suturing	No	Not reported

*D1*, First portion of the duodenum (aka duodenal bulb); *D2*, second portion of the duodenum; *OTSC*, over-the-scope clip; *TTSC*, through-the-scope clip.

A few technical hurdles exist during use of OTSCs and are summarized here. Having an en-face view of the defect before clip deployment is crucial. Therefore, turning the tip of the straight-viewing therapeutic gastroscope with the OTSC attached to it is usually required. This might prove challenging depending on the location of the defect in the duodenum. This generally requires a skilled assistant or a second endoscopist trained in OTSC deployment to manipulate the dual-pronged grasper to grab first one edge of the defect and then the other as the scope tip is deflected in the direction of the opposite side of the perforation. Such coordination and closure of a relatively wide and oblique defect may be practiced in the setting of an ex vivo model. The clinical impact for the patient resulting from timely successful deployment is clear. The cases we presented in this work highlight the importance of early recognition and treatment of post-ERCP and -EUS perforations to ensure the best outcome possible.

## Patient Consent

The authors have received appropriate patient consent for the publication of this article.

## Disclosure

*The following authors disclosed financial relationships: J. Cohen: Consultant for Olympus and Micro-Tech; stock owner in Virtual Health Partners, GI Windows, and Rom-Tech; owner of MD Medical Navigators; royalties from Wiley and Wolters Kluwer. G. B. Haber: Consultant for Olympus and Ovesco. All other authors disclosed no financial relationships.*

## Supplementary Material

Video 1 ▪▪▪
